# Up-regulation of *FOXN3-AS1* in invasive ductal carcinoma of breast cancer patients

**DOI:** 10.1016/j.heliyon.2021.e08179

**Published:** 2021-10-14

**Authors:** Samira Molaei Ramshe, Hamid Ghaedi, Mir Davood Omrani, Lobat Geranpayeh, Behnam Alipour, Soudeh Ghafouri-Fard

**Affiliations:** a Department of Medical Genetics, School of Medicine, Shahid Beheshti University of Medical Sciences, Tehran, Iran; b Urogenital Stem Cell Research Center, Shahid Beheshti University of Medical Sciences, Tehran, Iran; c Department of Surgery, Sina Hospital, Tehran, Iran; d Department of Laboratory Sciences, Faculty of Paramedicine, Yasuj University of Medical Sciences, Yasuj, Iran

**Keywords:** EMX2OS, FOXN3-AS1, GWAS, Breast cancer

## Abstract

Oncogenic and tumor-suppressive roles of long non-coding RNA make them an appropriate target for expression analysis in cancer studies. In this study, we selected two lncRNAs (EMX2OS and FOXN3-AS1) that are resided near the GWAS-identified SNPs for breast cancer (rs2901157 and rs141061110). These transcripts have been identified in different cancer types as either oncogenes or tumor suppressors. In the present investigation, we aimed to quantify the expression level of EMX2OS and FOXN3-AS1 in 44 breast cancer samples and normal adjacent tissues (ANCTs). The FOXN3-AS1 expression level was significantly increased in breast cancer samples compared with ANCTs (P value = 0.02), Also its amounts could distinguish two sets of samples with an accuracy of 70% (P value = 0.009). We have found an association between FOXN3-AS1 expression and tumor size (P value = 0.02). On the other hand, no significant differences were found in the EMX2OS expression level between two sets of samples (P value = 0.44); however, EMX2OS expression level has a significant association with the age of the patients (P value = 0.03). According to our result, FOXN3-AS1 can be demonstrated as a probable diagnostic marker in breast cancer so we suggest further functional studies to find the precise role of these lncRNAs in breast cancer progression.

## 1 Introduction

Breast cancer is the most common diagnosed cancer worldwide and the first cause of cancer-related death in women [1]. There are almost 21 different histological types of breast cancer with distinct incidence rates, causes, treatment options and survival rates [2]. Invasive ductal carcinoma (IDC) is the most common histological type, representing 75 percent of all invasive breast cancer diagnoses [3]. As a neoplasm, breast cancer is a heterogeneous disease and has both environmental and genetic risk factors like aging, ethnicity, lifestyle, and genetic factors [4].

A plethora of evidence suggests strong genetic components for breast cancer. In three percent of all breast cancer cases, mutations have been identified in a number of cancer-susceptibility genes. Recent studies have shown that single-nucleotide polymorphisms (SNPs) in number of genes can explain 18% of the inherited risk of breast cancer [5]. Interestingly, most of the significant SNPs in the genome-wide association studies (GWAS) reside in noncoding regions of the genome such as long non-coding RNAs (lncRNAs) [6]. LncRNAs are the largest group of non-coding RNAs in the human genome. Researches revealed that most of the lncRNAs show tissue-specific expression patterns and play key roles in a range of biologic processes including epigenetic regulation and modulation of gene expression at transcriptional and post-transcriptional levels. Moreover, recent data suggest important roles for lncRNAs in the modulation of oncogenic and tumor suppressor signaling pathways. Therefore, dysregulation of lncRNA expression could promote tumorigenesis process and progression of cancer [7]. For instance, Jang et al in 2014 have revealed ARA as an lncRNA in the intronic region of the X chromosome has a modulatory role in the MAPK signaling pathway and it is up-regulated in breast cancer [8]. Inspecting the GWAS catalog, we found a total of 1118 (as accessed in January 2020) breast cancer-associated SNPs resided in the lncRNA loci. In the present study we aimed to quantify expression level of two lncRNAs (EMX2OS and FOXN3-AS1) resided near the GWAS-identified SNPs for breast cancer (rs2901157 and rs141061110) in the breast cancer and normal adjacent tissues.

## 2 Materials and methods

### 2.1 Study population

Forty-five tissue samples from female patients with histopathological diagnosis of breast invasive ductal carcinoma were entered our study. They have no familial history and anticancer treatments before surgery (https://doi.org/10.1002/jcb.28629). All patients were informed about the assessment; they signed a consent form and filled out the questionnaire for demographic data collection. Tumor grades, estrogen receptor (ER) and progesterone receptor (PR), and other relevant parameters were recorded from histopathological reports. Both cancerous and adjacent non-cancerous breast tissues (ANCTs) were excised during mastectomy in the Sina and Farmanie hospitals in Tehran in 2020. We chilled samples on the liquid nitrogen for transferring to the Medical Genetics laboratory, Shahid Beheshti University of Medical Sciences to further processing. The study protocol was approved by the ethical committee of Shahid Beheshti University of Medical Sciences (IR.SBMU.MSP.REC.1398. 725).

### 2.2 LncRNAs selection

We queried the human genome to identify lncRNAs that resided in the flanking sequence of breast cancer-associated GWAS-SNP. We obtained a list of breast-cancer GWAS-SNP from the GWAS catalog (accessed on January 2020). An association block for each SNPs were defined, that included a stretch of ±50 Kb regarding the coordinate of GWAS-SNP. By intersecting each association block with human genome, we obtained a list of genes in these blocks. Further, genes under “Long non-coding RNA (lncRNA)” Ensembl annotation category were included for further analysis. Since most of new lncRNAs were poorly annotated, we had to consider the Ensembl Transcript Support Level (TSL) as a measure of molecule support level (Table 1). Based in these criteria for this research, we selected two lncRNAs, EMX2OS and FOXN3-AS1, for expression analysis. We selected EMX2OS and FOXN3-AS1 as our targets because they had the lowest acceptable P values among other lncRNAs in this list according to RNA data sets from TCGA. They also had an appropriate Ensembl Transcript Support Level (TSL = 1) as a measure of the molecule support level. The rs2901157 variant of EMX2OS has an association with breast cancer risk (P value: 2.00E-06) and rs141061110 resided in FOXN3-AS1 with P value 5.00E-06 [9].

**Table 1:** Data mining results for lncRNAs that resided within 50Kb from the breast cancer GWAS-SNPs. **GWAS:** Genome-wide association studies. **TSL:** Transcript Support Level.

Lnc RNA Name	GWAS-SNP	GWAS- P value	Trait	TSL	PMID
H19	rs217727	4.00E-14	Breast cancer	1	29059683
KANSL1-AS1	rs2532263	7.00E-13	Breast cancer	1	29059683
FOXN3-AS1	rs141061110	5.00E-06	Breast cancer	1	29059683
RUSC1-AS1	rs7524950	3.00E-09	Breast cancer	2	29059683
MAPT-AS1	rs62061734	8.00E-12	Breast cancer	2	29059683
LINC00886	rs78579487	4.00E-07	Breast cancer	2	29059683
HCG18	rs3094054	1.00E-07	Breast cancer	2	29059683
EGOT	rs6762644	2.00E-12	Breast cancer	1	23535729
EMX2OS	rs2901157	2.00E-06	Breast cancer	1	29059683
RAMP2-AS1	rs151329939	5.00E-09	Breast cancer	1	29059683
SEC16B	rs575908	3.00E-06	Breast cancer	1	29059683
GRIK1-AS1	rs458685	6.00E-06	Breast cancer	1	17903305
LINC00240	rs34546498	9.00E-10	Breast cancer	1	29059683
PROSER2-AS1	rs12358475	2.00E-06	Breast cancer (survival)	2	25526632
PSMD6-AS2	rs1053338	9.00E-09	Breast cancer	2	25751625
	rs3821902	3.00E-12	Breast cancer		29059683
CDKN2B-AS1	rs78545330	3.00E-06	BRCA1/2-negative high-risk breast cancer	1	30323354
	rs1011970	3.00E-08	Breast cancer		20453838
	rs1081165	2.00E-13	Breast cancer		27117709
	rs3057314	7.00E-25	Breast cancer		29059683
ADAMTS9-AS2	rs2030217	2.00E-06	Breast cancer specific mortality in breast cancer	1	30787463
MRPL23-AS1	rs217727	4.00E-14	Breast cancer	3	29059683
DNAJC27-AS1	rs1971136	5.00E-09	Breast cancer	1	29059683
CYP1B1-AS1	rs184577	4.00E-06	Breast cancer in BRCA2 mutation carriers	5	23544012
KIF9-AS1	rs9867461	2.00E-06	Breast cancer	2	29059683
AP4B1-AS1	rs11552449	2.00E-08	Breast cancer	2	23535729
	rs7513707	2.00E-11	Breast cancer		29059683
LINC00518	rs9348512	4.00E-08	Breast cancer in BRCA2 mutation carriers	1	23544012
LINC00266-1	rs6062356	3.00E-06	Breast cancer	1	29059683
CFLAR-AS1	rs182731523	8.00E-08	Breast cancer	1	29059683
AQP4-AS1	rs2307561	8.00E-18	Breast cancer	2	29059683
LINC00599	rs11786541	2.00E-06	Response to chemotherapy in breast cancer hypertensive cases (cumulative dose) (bevacizumab)	1	25117820
LMAN1L	rs6938	9.00E-08	Breast cancer	1	29059683
HCG9	rs3094146	7.00E-07	Breast cancer	1	29059683
MEG8	rs2295389	2.00E-06	Breast cancer	3	29059683

### 2.3 Quantitative real-time RT-PCR

Total RNA from paired tumoral and ANCTs samples was extracted by GeneAll® Hybrid-R™ 100 preps (Cat.No: 305-101; Seoul, South Korea) according to the manufacturer's protocol. For cDNA synthesis, we used SMOBIO ExcelRT™ Reverse Transcription Kit. Relative expressions of EMX2OS and FOXN3-AS1 were measured in tumoral versus ANCTs samples by using the *B2M* gene as normalizer. Table 2 shows the nucleotide sequences of targets and normalizer genes primers used in this study. We used RealQ Plus Master Mix Green, High Rox (AMPLICON, Odense, Denmark) for qPCR, and reactions were performed in duplicate in the ABI StepOne Plus. Ten μl of master mix was used in a final volume of 20 μl. The thermal cycling program was as follows: Initial activation at 95 °C for 15 min followed by 40 cycles of denaturation (95 °C for 5 s), annealing (60 °C for 20 s), and extension (72 °C for 20 s).

**Table 2:** Primers for lncRNAs qRT-PCR detection.

Gene name	Primer sequences	Primer length	Product length
EMX2OS	F: AATGCCACCTCTCTGCTTGACTG	23	160
	R:AACACCCTTAGACTTCCACACAATCC	26	
FOXN3-AS1	F:TGAGCCATCAATCATCCTTTCCTAAC	26	111
	R: GCCCATTTCTTCCACAGAGCAG	22	
B2M	F:AGATGAGTATGCCTGCCGTG	20	105
	R:GCGGCATCTTCAAACCTCCA	20	

### 2.4 Statistical analysis

EMX2OS and FOXN3-AS1 relative expression levels were estimated by using the E (Real-time PCR efficiencies) and the CT (cycle threshold) in both tumoral and ANCT samples where the B2M gene was used as the housekeeping gene for data normalization. The Prism software version 8 (GraphPad, San Diego, CA, USA) and the SPSS Statistical Software Package (version 18.0) were applied for statistical analyses. Data distribution normality was checked by Kolmogorov–Smirnov and Shapiro-Wilk tests. We used Mann-Whitney U to evaluate the differential expression of lncRNAs in two sample groups (Tumorals vs ANCTs). Moreover, Pearson's chi-square and Kruskal-Walis tests were used for evaluating the lncRNAs expression level association with clinical and demographic data. For estimating EMX2OS and FOXN3-AS1 diagnostic roles in tumoral samples, Receiver operating characteristic (ROC) curve analysis was used. P < 0.05 was considered significant in all statistical analyses.

### 2.5 Ethical approval

The informed consent was signed by the patients and all the study protocol was following the Hesinki Declaration. The Ethical Committee of Shahid Beheshti University of Medical Sciences (IR.SBMU.MSP.REC.1398.725) approves study procedures.

## 3 Results

### 3.1 lncRNAs in breast cancer association blocks

Table 1 summarized our data mining results for lncRNAs that resided within 50Kb from the breast cancer GWAS-SNPs.

### 3.2 Patients clinical and demographic characteristics

Paired samples (tumoral and non-tumoral) were obtained from 44 female patients. Clinical and demographic data have been gathered from either clinical, para clinical tests before and after surgery or questionnaires filled by patients. Table 3 shows the patients’ data summary.

**Table 3:** General demographic and clinical data of patients.

Variable	Value
Age (years) (mean ± SD)	51.22 ± 12.91
Menarche age (years) (mean ± SD)	13.02 ± 1.6
Menopause age (years) (mean ± SD)	48.2 ± 16
First pregnancy age (years) (mean ± SD)	16.68 ± 9.2
Breast feeding duration (months) (mean ± SD)	39.20 ± 36.39
Positive family history for cancer (%)	31.81
Cancer stage (%)	
I	31.81
II	27.27
III	34.09
IV	6.81
Histo grade (%)	
I	29.54
II	40.9
III	29.54
Mitotic rate (%)	
I	38.63
II	47.72
III	13.63
Tumor size (%)	
<2 cm	31.81
> = 2 cm, <5 cm	63.63
> = 5 cm	4.54
Estrogen receptor (%)	
Positive	77.27
Negative	22.72
Progesterone receptor (%)	
Positive	70.45
Negative	29.54
Her2/neu expression (%)	
Positive	45.45
Negative	54.54
Ki67 expression (%)	
Positive	81.81
Negative	18.19

### 3.3 EMX2OS and FOXN3-AS1 differential expression in breast cancer samples vs. ANCTs

**Figure 1:**
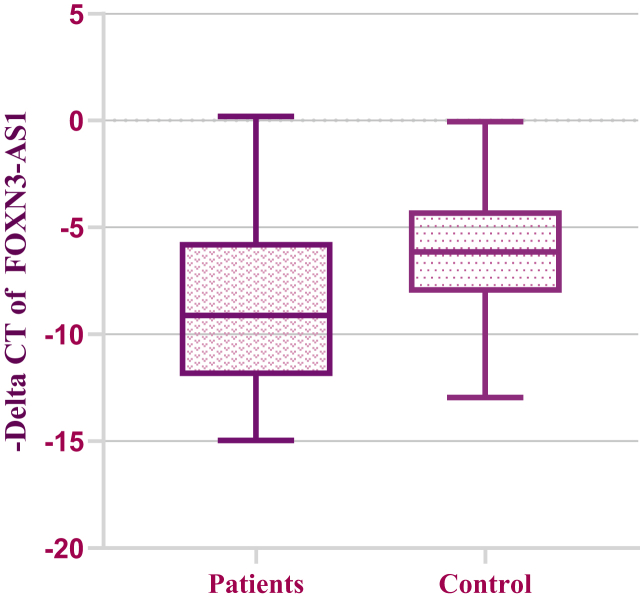
Relative expression of FOXN3-AS1 in breast cancer samples (n = 44) and ANCTs (n = 44) as described by –delta CT Values (CT B2M - CT target gene).

**Figure 2:**
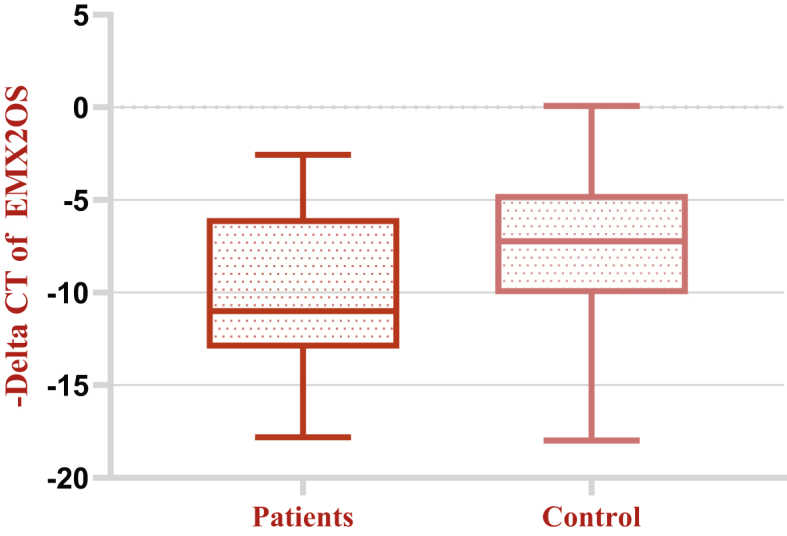
Relative expression of EMX2OS in breast cancer samples (n = 44) and ANCTs (n = 44) as described by –delta CT Values (CT B2M - CT target gene).

According to statistical analysis, FOXN3-AS1 was shown to be considerably up-regulated in breast cancer samples in comparison with ANCTs (P value = 0.02). Nevertheless, EMX2OS expression analysis could not reveal statistically significant differences between tumoral tissues and ANTCs (P value = 0.44). –delta CT was used to illustrate the relative expression of FOXN3-AS1 and EMX2OS in two sample groups in Figures 1 and 2.

### 3.4 Correlation of EMX2OS and FOXN3-AS1 expression with clinical and demographic characteristics

After measuring ΔCt medians of genes expression and interquartile range, a significant association was identified between FOXN3-AS1 expression and tumor size (Table 4). FOXN3-AS1 was more frequently up-regulated in breast cancer patients who have tumor size >2 compared with patients with tumor size ≤2 (P value = 0.02). Furthermore, EMX2OS expression level has a significant association with the age of the patients (P value = 0.03). There was not any other significant association between FOXN3-AS1 or EMX2OS expression levels and other characteristics.

**Table 4:** FOXN3-AS1 and EMX2OS expression levels (medians of ΔCt and interquartile range) according to the demographic and clinical data of the patients. P values were obtained by Mann–Whitney U test.

Characteristics	N	FOXN3-AS1	P value	EMX2OS	P value
Age			0.24		0.03
≤55	31	9.54 (6.66–11.77)		11.77 (9.06–12.62)	
>55	13	9.41 (7.12–12.55)		8.40 (6.21–11.12)	
Stage			0.30		0.41
1,2	27	12.50 (11.42–13.32)		10.75 (6.43–11.96)	
3,4	17	7.58 (4.36–10.45)		6.98 (5.19–8.99)	
Mitotic rate			0.86		0.86
1	20	9.01 (6.35–11.35)		7.54 (5.42–12.14)	
2, 3	24	4.81 (3.33–7.68)		7.15 (4.27–10.05)	
Histological grade			0.68		0.81
1	13	8.84 (5.32–12)		10.94 (8.66–13.33)	
2, 3	31	7.59 (4.70–10.71)		8.93 (5.42–12.12)	
Tumor size			0.02		0.18
≤2	18	5.83 (2.49–9.23)		6.50 (4.33–11.86)	
>2	26	11.32 (9.61–12.25)		10.55 (8.33–12.19)	
ER Status			0.45		0.57
Positive	34	7.59 (3.91–11.22)		8.56 (5.51–12.13)	
Negative	10	10.86 (8.45–12.01)		10.55 (5.83–12.05)	
PR Status			0.85		0.41
Positive	31	8.22 (4.26–11.17)		8.72 (5.59–12.14)	
Negative	13	5.71 (3.66–8.46)		6.87 (4.52–9.74)	
HER2 Status			0.90		0.72
Positive	20	7.58 (2.53–11.77)		9.92 (4.33–12.04)	
Negative	24	8.84 (5.74–10.64)		8.17 (5.52–11.85)	

### 3.5 ROC curve analysis

**Figure 3:**
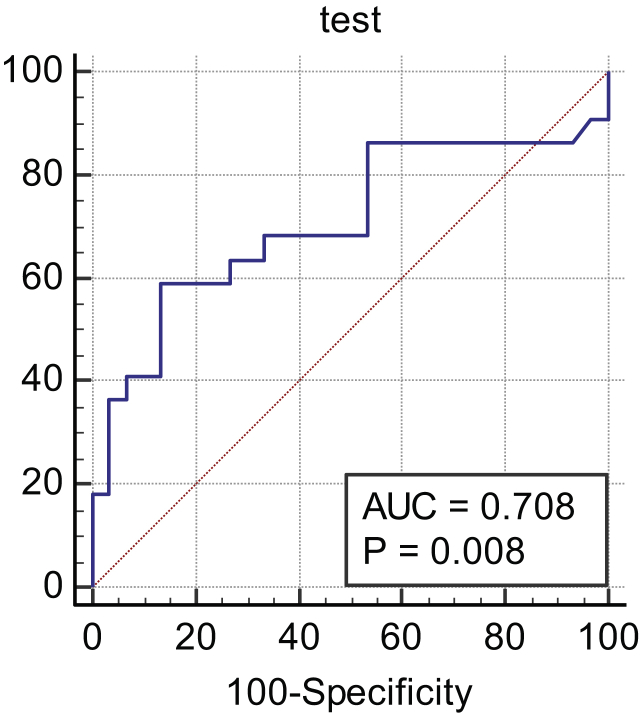
The results of ROC curve analysis for evaluating FOXN3-AS1 diagnostic power for breast cancer. ROC, Receiver operating characteristic.

ROC curve analysis was performed for FOXN3-AS1, which has a differential expression level in tumoral samples versus ANCTs. Detailed information on ROC curve analysis for evaluating FOXN3-AS1 as a diagnostic biomarker is shown in Table 5 and Figure 3.

**Table 5:** The results of ROC curve analysis.

	Estimate criterion	AUC	Youden index	Sensitivity	Specificity	P value
FOXN3-AS1 transcript levels	>8.770	0.70	0.45	59.09	86.67	0.009

## 4 Discussion

Breast cancer is one of the most common and complicated cancers worldwide. Recently, lncRNAs have been shown to have causative roles in different cancer types including breast cancer [10, 11]. Although precise roles of lncRNAs in breast cancer have not been completely defined yet, they have been identified as either oncogenes or tumor suppressor genes depending on their expression manner during tumor growth [12].

Different studies have used multiple approaches to choose target non-coding RNA that may have an association with breast cancer. Choosing lncRNAs from frequently mutated regions in breast cancer [13] and those targeting breast cancer signaling pathways [14] were some common approaches that have been used before. In this study, we have considered the lncRNAs, which are located in breast cancer GWAS loci. We suggest that lncRNAs in the Table 1 might be implicated in the breast cancer development. However, this speculation needs to be further investigated.

EMX2OS and FOXN3-AS1 were chosen from a list of 30 lncRNAs that were located near breast cancer-associated SNPs. We have recently used this approach to select lncRNAs being involved in type 2 diabetes [15, 16, 17]. Consistent with our findings, transcribed ultraconserved regions (T-UCRs) as a group of lncRNAs conserved in numerous species have been found to be commonly located in the cancer-related regions [18, 19]. Similarly, several SNPs within lncRNAs regions have been demonstrated to be associated with breast cancer risk. For instance, some breast cancer risk variants have been demonstrated to target GABPB1-AS1 in INQUISIT and eQTL analyses [20]. Moreover, other risk-associated variants have been detected in the regions of two T-UCRs, namely uc.184 and uc.313 [20]. Another study has reported a novel breast cancer-related risk variant within in lncHSAT164, an up-regulated lncRNA in breast cancer samples and cell lines [21].

In our study, FOXN3-AS1 showed up-regulation in breast cancer samples in comparison with control samples. Also, in assessing the correlation of lncRNAs expression with clinical and demographic characteristics, we recognized a significant association between tumor size and FOXN3-AS1 expression.

FOXN3 is a member of the FOX gene family which act as transcription factors. This gene is expressed in most of the human tissues and has critical roles in cell growth, cell differentiation and tumorigenesis. Although FOXN3 dysregulation has been identified in different types of cancers such as liver and mouth carcinomas, glioblastoma, and Hodgkin's lymphoma [22, 23, 24], the exact molecular mechanism of its contribution in these cancers needs to be clarified. In breast cancer, Li et al have identified a transcription repressor role for FOXN3, which leads to repressing the transcription of FOXN3-NEAT1-SIN3A complex downstream genes, thus enhancing metastasis of breast cancer in vivo [25]. FOXN3 has two antisense transcripts namely FOXN3-AS1 and FOXN3-AS2. FOXN3-AS2 has been suggested to contribute in esophageal cancer and lung adenocarcinoma [26, 27]. To the best of our knowledge, dysregulation of FOXN3-AS1 is only identified in non-small-cell lung carcinoma (NSCLC). Authors have reported downregulation of FOXN3-AS1 in squamous cell carcinoma samples [28]. This expression pattern is in contrast with the results of the current study. Therefore, more functional investigations are needed to elucidate the exact role of FOXN3-AS1 in the tumorigenesis.

In contrast with the previously reported studies in various cancers [29, 30, 31, 32, 33] and location of EMX2OS in rs2901157 locus, we could not find different expression levels for EMX2OS in invasive ductal breast carcinoma samples versus ANCTs. EMX2OS is an anti-sense transcript and regulator of the EMX gene, which is a transcription factor. Although EMX2OS was previously identified to be expressed only in the central nervous system [34, 35], recent studies have shown its expression in different types of malignancies. For instance, overexpression of EMX2OS was reported in gastric cancer [29] and ovarian cancer [30] resulting in the enhancement of proliferation and invasion of cancer cells. On the other hand, down-regulation of this lncRNA has been identified in classical papillary thyroid cancer [31] and prostate cancer [32]. Wang et al. have recently revealed the negative regulatory role of EMX2OS in the proliferation and invasion of prostate cancer cells [32, 33]. Besides, Tang et al. have introduced EMX2OS as a novel diagnostic biomarker for recurrent laryngeal cancer and recurrence-free survival time of patients [33]. However, in our study, EMX2OS did not demonstrate differential expression levels in cancer cells versus ANCTs. However, EMX2OS expression level was significantly associated with the age of the patients. The small sample size in our project or the distinctive role of EMX2OS in breast cancer versus other cancers can be possible reasons for this contrary result.

Furthermore, we evaluated the diagnostic power of FOXN3-AS1 through ROC curve analysis. The obtained P value, the area under curve (AUC), sensitivity, and specificity illustrate the appropriate diagnostic power of FOXN3-AS1 up-regulation in breast cancer samples. Verification of the diagnostic power of FOXN3-AS1 needs further studies in larger samples sizes.

## 5 Conclusion

To conclude, we identified FOXN3-AS1 up-regulation in invasive ductal carcinoma type of breast cancer samples. In addition, this study demonstrates an association between FOXN3-AS1 expression levels with tumor stages. According to our investigation, this lncRNA can be as a probable diagnostic marker in breast cancer. In the case of EMX2OS, despite previous studies in other malignancies, we could not find a significant difference in its expression between tumor and control samples. We suggest conduction of upcoming studies with larger sample sizes and incorporation of other breast cancer histological subtypes to clarify the precise roles of these lncRNA in breast cancer progression.

## Declarations

### Author contribution statement

Samira Molaei Ramshe: Performed the experiments; Wrote the paper.

Mir Davood Omrani, Lobat Geranpayeh, Behnam Alipour and Soudeh Ghafouri-Fard: Conceived and designed the experiments.

Hamid Ghaedi: Conceived and designed the experiments; Analyzed and interpreted the data; Wrote the paper.

### Funding statement

This work was supported by 10.13039/501100005851Shahid Beheshti University of Medical Sciences.

### Data availability statement

Data will be made available on request.

### Declaration of interests statement

The authors declare no conflict of interest.

### Additional information

No additional information is available for this paper.
